# Association Study of Val66Met Polymorphism in Brain-Derived Neurotrophic Factor Gene with Clozapine-Induced Metabolic Syndrome: Preliminary Results

**DOI:** 10.1371/journal.pone.0072652

**Published:** 2013-08-13

**Authors:** Yi Zhang, Meijuan Chen, Zhiguo Wu, Jun Chen, Shunying Yu, Yiru Fang, Chen Zhang

**Affiliations:** 1 Schizophrenia Program, Shanghai Mental Health Center, Shanghai Jiao Tong University School of Medicine, Shanghai, China; 2 Division of Mood Disorders, Shanghai Mental Health Center, Shanghai Jiao Tong University School of Medicine, Shanghai, China; 3 Department of Genetics, Shanghai Mental Health Center, Shanghai Jiao Tong University School of Medicine, Shanghai, China; Kunming Institute of Zoology, Chinese Academy of Sciences, China

## Abstract

The prevalence of the metabolic syndrome (MetS) is higher among patients receiving atypical antipsychotics (AAPs) treatment, and even among AAPs, treatment with clozapine has been shown to be associated with a higher long-term incidence rate of MetS. Likewise, brain-derived neurotrophic factor (BDNF) deficiency has been reported to result in metabolic traits, such as increased food intake, hyperphagia and obesity, etc. In this study, we hypothesized that a functional polymorphism (Val66Met) in the BDNF gene may confer susceptibility to clozapine-induced MetS, potentially in a sex-specific manner, since an interaction between Val66Met polymorphism and sex was observed in our previous studies. A total of 199 schizophrenia patients being treated with clozapine were divided into two groups, MetS and non-MetS, based on the diagnostic criteria of the National Cholesterol Education Program's Adult Treatment Panel III. We genotyped the Val66Met polymorphism, and measured the serum levels of fasting glucose (GLU), triglyceride (TG) and high density lipoprotein cholesterol (HDL). There was a trend indicating a significant association between the homozygous Met/Met genotype and MetS in male patients (OR = 2.39; 95% CI: 1.05–5.41; *p* = 0.039; corrected *p* = 0.078). Among the six risk factors listed in the ATPIII criteria, we found a significant association between fasting GLU levels and Val66Met polymorphism in males (*p* = 0.005; corrected *p* = 0.03), but not in females (*p* = 0.65). Post-hoc analysis in males revealed that the Met/Met carriers had significant higher levels of fasting GLU than those with Val/Val or Val/Met genotypes (*p* = 0.007; corrected *p* = 0.042 and *p* = 0.002; corrected *p* = 0.012, respectively). In conclusion, we observed a weak association between the Val66Met polymorphism and clozapine-induced MetS in a sex-specific manner. While preliminary, such findings prompt further, large-scale longitudinal studies to replicate these findings.

## Introduction

Antipsychotic drugs have been widely used to treat psychosis, particularly among patients with schizophrenia and bipolar disorder. Antipsychotics are likewise increasingly becoming accepted for managing of non-psychotic disorders. Over the past decade, atypical antipsychotics (AAPs) have been approved by the U.S. Food and Drug Administration and are now more frequently prescribed than typical antipsychotics. However, clinical observations have indicated that treatment with AAPs are associated with obesity and other components of metabolic syndrome (MetS), particularly abnormal glucose and lipid metabolism [1]. In 2003, the U.S. Food and Drug Administration (FDA) issued a warning associating AAPs with a connection to increased risk for diabetes and hyperglycemia.

Clozapine is an atypical antipsychotic often used for its demonstrated superiority in the treatment of refractory schizophrenia [2], [3]. However, clozapine exhibited a higher long-term incidence rate of MetS (up to 58.3%) as compared with other AAPs, using the National Cholesterol Education Program's Adult Treatment Panel III (ATP III) criteria [4], [5]. Accordingly, investigating the mechanisms that contribute to the high occurrence of clozapine-induced MetS may help facilitate the identification and clinical management of this significantly adverse effect.

Obesity, a status characterized by excessive adipose tissue, is one of the most important components of MetS [6]. In rodents, models of brain-derived neurotrophic factor (BDNF) disruption all exhibited increased food intake, obesity, and hyperphagia [7], [8], whereas calorie restriction in heterozygous *BDNF* ^+/−^ mice can increase expression of BDNF and reduce obesity [9], [10]. In humans, BDNF was found to be associated with eating disorders, such as restrictive anorexia nervosa [11], as well as antipsychotic-induced body weight gain [12], [13], [14], [15]. In a recent pharmacogenetic study, BDNF was reported to regulate the clozapine treatment response [16], suggesting that BDNF may be a pharmacological target of clozapine. Given these findings, it seems plausible that a deficiency in BDNF may be a potential biological mechanism that underlies clozapine-induced MetS.

At the molecular level, a common functional polymorphism of *BDNF*, leading to a valine to methionine substitution at codon 66, is known to alter the intracellular trafficking and activity-dependent secretion of BDNF [17]. Recently, the Val66Met polymorphism was identified as a risk factor for metabolic traits, such as obesity and insulin resistance in several human populations [12], [13], [14], [18], [19], [20], [21].

Based on the findings from abovementioned studies, we hypothesized that the *BDNF* Val66Met polymorphism may confer susceptibility to clozapine-induced MetS. To test this potential association, we conducted a pharmacogenetic study to investigate the association between the Val66Met polymorphism and MetS in a population of Han Chinese under long-term clozapine treatment. Taking into consideration the interaction between the Val66Met polymorphism and sex observed in our previous studies [22], [23], we also aimed to evaluate potential differences in the effect of this variant on males and females.

## Methods

### Ethics Statement

This study was reviewed and approved by the ethics committee of the Shanghai Mental Health Center. All participants provided written informed consent prior to inclusion in this project, and were treated in accordance with the Declaration of Helsinki. The assessment of participants’ capacity to provide consent was based on their (1) ability to communicate a reasoned choice regarding participation; (2) ability to understand relevant information regarding the study, including consequences of participation for the participant’s own situation (such as health condition) and consequences of the alternatives to participation; (3) ability to comprehend the nature of the situation and its likely consequences; and (4) ability to manipulate information rationally. Next of kin, carer takers, or guardians consented on the behalf of participants whose capacity to consent was compromised.

### Participants

A total of 199 unrelated Han Chinese schizophrenia patients (143 males and 56 females, aged 55.0±7.4 and 55.9±5.2, respectively) were recruited from the Inpatient Psychiatry Unit at Shanghai Mental Health Center, Shanghai Jiao Tong University School of Medicine. The inclusion criteria for patients consisted of six conditions: (1) patients had been diagnosed with schizophrenia according to the DSM–IV, with the diagnoses either made or reviewed by experienced psychiatrists; (2) patients were free from MetS before receiving clozapine, based on the medical records; (3) patients were receiving clozapine treatment alone or in conjunction with typical antipsychotics, but not atypical ones as other atypical antipsychotics (e.g. olanzapine, quentipine) may potentially enhance the risk of MetS [2]; (4) patients had been receiving clozapine for more than 24 months [24], [25]; (5) patients had maintained a stable condition for more than six months before entry into the study; and (6) patients had no other diagnosed psychiatric disorders aside from schizophrenia.

### Diagnosis of clozapine-induced MetS

A cross-section assessment of metabolic parameters was performed to determine the prevalence of MetS based on the ATPIII definition, which comprises the best criteria for diagnosing MetS in a Chinese population [26]. MetS was diagnosed in the presence of any three of the following: (1) a waist circumference ≥90 cm in Chinese men and ≥80 cm in Chinese women [27]; (2) triglyceride (TG) ≥1.7 mmol/l; (3) high density lipoprotein cholesterol (HDL) <1.0 mmol/l in men and <1.3 mmol/l in women; (4) blood pressure ≥130/85 mmHg; or (5) fasting glucose≥5.6 mmol/l [28].

### Metabolic parameters analysis

Waist circumference was measured between the lower rib margin and the iliac crest, after a normal expiratory breath. Serum fasting GLU, TG, and HDL levels were measured using an automatic Biochemical Analyzer (HITACHI 7170A, Hitachi, Ltd, Tokyo, Japan). Overnight fasting blood samples were drawn between 7:00 and 7:30 a.m. from all patients.

### Genotyping

The Val66Met polymorphism, also known as rs6265 (G/A), is located at Chr.11:27679926 based on National Center for Biotechnology Information database (http://www.ncbi.nlm.nih.gov/projects/SNP/snp_ref.cgi?rs=6265). Indentified from HapMap-HCB (Han Chinese in Beijing) database, the more common A allele of rs6265 encodes the Met, while the G allele encodes Val.

In this study, the Val66Met polymorphism was amplified independently by PCR and genotyped by direct sequencing using an ABI PRISM 3730 Genetic Analyzer (Perkin-Elmer Applied Biosystems). Genotyping was carried out according to the method described previously by Zhang et al. [29]. PCR amplification was performed in a volume of 25 µL containing a primer pair (Forward: 5′-AAACATCCGAGGACAAGGTG-3′; Reverse: 5′-CCTCATGGACATGTTTGCAG-3′). PCR primers were also used for sequencing. Sequencing results were handled by the DNAStar (DNAstar Inc. USA) and the original sequencing chromatograms of each sample were further checked manually.

### Statistical analysis

SPSS 17.0 (SPSS Inc., Chicago, IL, USA) was used to perform either the *t*-test or chi-square test in order to compare demographic characteristics between the MetS and non-MetS groups. The online program SHEsis (http://analysis.bio-x.cn) [30] was used to test Hardy-Weinberg equilibrium. Odds ratios were used to measure the association of MetS risk with the alleles and genotypes of the Val66Met polymorphism. Unconditional logistic regression models were used to obtain maximum-likelihood estimates of the odds ratios (ORs) and their 95% confidence intervals (CIs). Analysis of the relationship between the individual metabolic parameters (dependent variable) and the Val66Met genotypes (independent variable) was performed with ANCOVA. Variables that affected metabolic parameters (i.e. age, sex, duration of clozapine treatment, clozapine doses) were included as covariates. To correct for multiple testing using the Bonferroni test, corrected *p* values were set at an uncorrected *p* value multiplied by *k* (independent significance tests). All *p* values are two-tailed, and *p* values below 0.05 were considered statistically significant after Bonferroni correction.

## Results

MetS was found in 86/199 of the patients (43.2%), with 40.0% prevalence (57/143) in males and 51.8% (29/56) in females. Results showed no difference between the MetS and non-MetS groups in terms of age, sex, duration of clozapine treatment, and clozapine dose. Patients with MetS had notably higher BMI, waist circumference, fasting GLU, fasting TG, fasting HDL, SBP and DBP than those without MetS. There was no significant difference in either allele or genotype frequencies between the schizophrenia patients with and without MetS. Further analyses based on sex stratification revealed a marginal association of the homozygous Met/Met genotype with MetS in male patients (OR = 2.39; 95% CI: 1.05–5.41; *p* = 0.039; corrected *p* = 0.078) (Table 1).

**Table 1:** Distribution of Val66Met genotype and allele in schizophrenic patients with or without MetS.

	Genotype distribution (%)				Odds ratio (95% CI)	*p* c	Allele (%)			*p* c
MetS group	n	Val/Val	Val/Met	Met/Met			n	Val	Met	
Total (Males, Females)	86	20 (23.3)	45 (52.3)	21 (24.4)	1.71 (0.84–3.45) ^a^	0.15	172	85 (49.4)	87 (50.6)	0.52
					0.89 (0.45–1.75) ^b^	0.73				
Males	57	11 (19.3)	29 (50.9)	17 (29.8)	2.39 (1.05–5.41) ^a^	0.039	114	51 (44.7)	63 (55.3)	0.10
					1.35 (0.59–3.07) ^b^	0.54				
Females	29	9 (31.0)	16 (55.2)	4 (13.8)	0.70 (0.17–2.95) ^a^	0.73	58	34 (58.6)	24 (41.4)	0.19
					0.27 (0.07–1.17) ^b^	0.10				
non-MetS group										
Total (Males, Females)	113	24 (21.2)	71 (62.8)	18 (15.9)			226	119 (52.7)	107 (47.3)	
Males	86	21 (24.4)	52 (60.5)	13 (15.1)			172	94 (54.7)	78 (45.3)	
Females	27	3 (11.1)	19 (70.4)	5 (18.5)			54	25 (46.3)	29 (53.7)	

The Odds ratio was calculated for MetS group homozygous for Met allele ^a^ (Met/Met vs. Val/Val+Val/Met), and homozygous or heterozygous for Met allele ^b^ (Met/Met+Val/Met vs Val/Val).

c *p* values were not corrected for multiple test.

**Figure 1:**
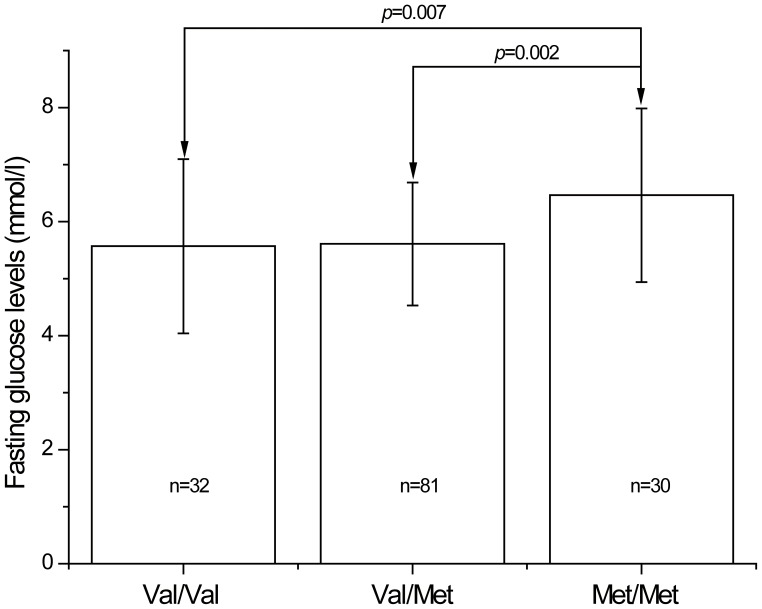
Fasting levels of GLU in males classified according to the *BDNF* Val66Met genotypes. Each column represents the mean±SD. Met/Met homozygous individuals had significantly higher levels of fasting GLU than those with Val/Val or Val/Met genotypes (corrected *p* = 0.042 and corrected *p* = 0.012, respectively).

Among the six risk factors listed in the ATPIII criteria, a significant association was found between fasting GLU levels and the Val66Met polymorphism, but the significance did not survive after Bonferroni correction (*F*_6, 192_ = 2.24, *p* = 0.04; corrected *p* = 0.24) (Table 2). The mean ± standard deviation fasting GLU levels (mmol/l) of Val/Val, Val/Met and Met/Met carriers were 5.7±1.4, 5.6±1.1 and 6.2±1.5, respectively. After stratification based on sex, results did indicate a significant association between fasting GLU levels and Val66Met polymorphism in males (*p* = 0.005; corrected *p* = 0.03), but not in females (*p* = 0.65) (Table 3). Post-hoc analysis in males further revealed that the Met/Met carriers had significantly higher levels of fasting GLU than those with Val/Val or Val/Met genotypes (*p* = 0.007; corrected *p* = 0.042 and *p* = 0.002; corrected *p* = 0.012, respectively) (Figure 1).

**Table 2:** BDNF Val66Met polymorphism and individual parameters of 199 subjects.

	Val/Val (n = 44)	Val/Met (n = 116)	Met/Met (n = 39)	*p* a
Waist circumference (cm)	88.0 (5.9)	86.9 (6.4)	87.0 (6.9)	0.76
Fasting GLU (mmol/l)	5.7 (1.4)	5.6 (1.1)	6.2 (1.5)	0.04
Fasting TG (mmol/l)	1.6 (0.8)	1.6 (0.9)	1.3 (0.5)	0.22
Fasting HDL (mmol/l)	1.0 (0.5)	1.2 (0.5)	1.0 (0.3)	0.11
SBP (mm Hg)	119.4 (9.1)	118.7 (10.0)	120.0 (9.9)	0.89
DBP (mm Hg)	77.8 (5.3)	76.8 (5.7)	76.8 (6.3)	0.36

Data were presented as mean (S.D.).

a *p* values were adjusted for age, gender, duration of clozapine treatment and drug dose, and not corrected for multiple test.

**Table 3:** BDNF Val66Met polymorphism and individual parameters in males and females.

	Males, mean (S.D.)				Females, mean (S.D.)			
	Val/Val (n = 32)	Val/Met (n = 81)	Met/Met (n = 30)	*p* a	Val/Val (n = 12)	Val/Met (n = 35)	Met/Met (n = 9)	*p* a
Waist circumference	89.1 (6.0)	89.1 (4.9)	88.7 (5.4)	0.87	85.0 (4.6)	81.9 (6.7)	81.2 (7.3)	0.13
Fasting GLU (mmol/l)	5.6 (1.5)	5.6 (1.1)	6.5 (1.5)	0.005	5.9 (1.2)	5.6 (1.2)	5.2 (0.8)	0.65
Fasting TG (mmol/l)	1.6 (0.9)	1.5 (0.9)	1.3 (0.6)	0.52	1.6 (0.5)	1.8 (1.1)	1.1 (0.3)	0.09
Fasting HDL (mmol/l)	1.1 (0.4)	1.2 (0.4)	1.0 (0.4)	0.10	1.0 (0.6)	1.2 (0.5)	1.3 (0.3)	0.07
SBP (mm Hg)	119.5 (10.1)	118.3 (10.2)	119.8 (10.1)	0.82	119.2 (6.0)	119.4 (9.8)	120.6 (8.8)	0.92
DBP (mm Hg)	77.8 (5.4)	76.8 (5.9)	76.5 (6.7)	0.15	77.9 (5.4)	76.9 (5.4)	77.8 (5.1)	0.56

a *p* values were adjusted for age, duration of clozapine treatment, and drug dose, and not corrected for multiple test.

## Discussion

BDNF is a member of the neurotrophin family, which regulates various neurodevelopmental processes, such as neuronal differentiation, neurite outgrowth and neuronal survival [31]. Altered BDNF signaling has been shown to involved in a variety of peripheral and central nervous system disorders, including dementia, amyotrophic lateral sclerosis, depression and schizophrenia, etc [32]. A recent study implicated BDNF in the control of GLU, lipid and antioxidant metabolism [33], which is considered an anorexigenic signal in the central control of food intake [34]. By contrast, heterozygous knock-out mice for *BDNF* showed hyperphagia and obesity [35]. BDNF then seems to play similar roles in neuronal development and the regulation of energy homeostasis [36], and accordingly has been conceptually viewed not only as a neurotrophin, but also a metabotrophic factor [33].

Several studies reported that serum concentrations decreased in subjects with MetS, and that a supplement of BDNF could reduce body weight [37], [38], [39], [40]. However, opposing results have also been reported [6], [41]. The Met allele has been known to significantly reduce activity-dependent secretion of BDNF as compared with the Val allele [17].The Met/Met genotype thus results in considerable low-activity of BDNF protein. In the present study, we observed a trend towards an association of the Met/Met genotype with clozapine-induced MetS in males. This finding is in agreement with the association between low BDNF concentration and MetS. Another major finding of this study is the association of the Val66Met polymorphism with clozapine-induced increased fasting GLU levels. BDNF has been reported to influence fasting GLU levels and insulin sensitivity [36], and decreased levels of the serum BDNF were found in the patients with MetS and type 2 diabetes mellitus [37], [42]. Mice with *BDNF* haploinsufficiency exhibited obesity and elevated levels of GLU [10], whereas systemic peripheral administration of BDNF contributed to the improvement of GLU metabolism and prevented the development of diabetes [43], [44]. In humans, there is a significant correlation between increased GLU levels and the *BDNF* Met allele in the general population [45]. Insulin is critical for the body’s use of GLU as energy, and insulin resistance (IR) is a condition in which the body produces insulin but does not use it effectively, leading to increased levels of GLU. Clinical and preclinical studies have both documented that clozapine can result in marked IR [46]. Burghardt et al. reported that the *BDNF* Met allele alone and in combination with AAP medications is associated with higher IR values [21]. Similarly IR is well established as the major pathogenic feature of MetS [47]. Taken together, we assume that the *BDNF* Val66Met genotype confers susceptibility to MetS by decreasing insulin action in the peripheral tissues.

In this study, we found that the impact of the Met/Met genotype on MetS and increased levels of GLU is only seen in male patients. To our best knowledge, this is the first study to evaluate the sexual variation in the effects of the Val66Met polymorphism on MetS. Our previous studies also indicated that the Val66Met polymorphism may have sex-specific characteristics [22], [23]. A recent preclinical study showed that BDNF heterozygous mice have complex brain region-specific changes in neurotrophins and their receptors differ gender-specifically [48]. This finding suggested that BDNF-TrkB signaling may be controlled in a sex-specific manner. Therefore, our findings in the present study imply that the effects of BDNF on MetS may be dependent on sex. The following question then arises naturally: do sex steroids contribute to this effect? Further studies are required for clarification, given the limited sample size and scope of this study.

The strength of this study is that the patients recruited for the study were all subject to long-term hospitalization, under clozapine treatment, with the maximum control over drug compliance, and receiving the same amounts of daily diet and exercise. Alongside these strengths, however, we would be remiss in not noting some marked limitations to this study. First, the size of our sample is small, especially for the purposes of sex stratification, and accordingly our findings should be viewed as preliminary until replicated and independently verified. Second, our subjects are chronic patients, and other AAP treatment prior to this study may have already influenced the risk for MetS, lessening the effects attributable to clozapine that the patients are currently being treated with. Third, patients’ baseline metabolic parameters prior to clozapine treatment and their previous antipsychotic agents were unknown, which may potentially confound the results obtained in this study. Lastly, the ideal pharmacogenetic study design is a longitudinal, prospective, randomized and parallel-control clinical trial. However, our study was cross-sectionally designed rather than longitudinally, therefore we cannot affirm that some subjects in the non-MetS group did not develop MS after the investigation. To avoid the negative consequences of this potential bias, it is important to replicate this data using larger studies that are better designed to find conclusive and not simply suggestive evidence of the associations that we noted in the present study.

In summary, in this study we tested for the first time the relationship between the *BDNF* Val66Met polymorphism and MetS in patients with schizophrenia under long-term clozapine treatment. We concluded that BDNF appears to have a weak association with clozapine-induced MetS, and this effect is only evident in male patients. Large-scale longitudinal studies should be conducted to replicate these findings and offer more conclusive evidence.
